# Wearable Use, Engagement, and Best Practices: Lessons Learned From the Virtual My Healthy Brain Trial

**DOI:** 10.1093/geroni/igaf122.2815

**Published:** 2025-12-31

**Authors:** Makenna Law, Mallika Saksena, Ryan Mace

**Affiliations:** Massachusetts General Hospital, Boston, Massachusetts, United States; Massachusetts General Hospital, Boston, Massachusetts, United States; Massachusetts General Hospital, Boston, Massachusetts, United States

## Abstract

Physical activity and sleep are key lifestyle behaviors for promoting healthy aging and dementia risk reduction. Wearable activity monitors are frequently employed in research settings as an effective and valid method for objectively assessing activity and sleep. Although previous studies have investigated validity and usability for older adults, more guidance is needed on evidence-based strategies for increasing the adoption and adherence of digital health technologies embedded in behavioral dementia prevention trials. We conducted a remote randomized controlled trial with older adults (60+) with subjective cognitive decline and lifestyle risk factors comparing the 8-week My Healthy Brain program with an attention- and dose-matched educational control. We enrolled 49 older adults (M = 72, SD = 7.41; 86% female; 61% white). Participants set up a Garmin Vívosmart 5 virtually with study team assistance and were instructed to wear for baseline to post-test (9 weeks) to track step count and total sleep. Adherence surpassed our a priori feasibility benchmark, which required 70% of participants to achieve 5 of 7 days per week at a minimum of 10 hours per day (Unadjusted adherence = 78%, 91% after removing Garmin errors). Our presentation will share effective strategies to promote adherence and lessons learned such as establishing clear expectations, device use training over Zoom, addressing accessibility challenges, linking data to personal relevance, and troubleshooting Garmin errors. The feasibility data and adherence strategies will inform the design of a subsequent NIA Stage 2 trial testing the efficacy of My Healthy Brain to improve physical activity and sleep outcomes assessed via Garmin.

